# Supplementary material for: Undergraduates’ lifestyle and Suboptimal Health Status (SHS): A cross-sectional study in the Ha’il region of Saudi Arabia
Source: PLoS One. 2025 Jan 24;20(1):e0317127. doi: 10.1371/journal.pone.0317127 (PMC11761099; doi:10.1371/journal.pone.0317127)
Supplement: Attachment — Submitted filename: Response 2 to reviewers.pdf [file pone.0317127.s002.pdf]

Response to Reviewers comments

I as a corresponding author, thank you for providing us a chance to address the comments. In response to the comments, we have made necessary amendments in the manuscript to provide additional details regarding participant consent. In the ethics statement in the Methods and online submission information. We have also attached the Ethical Approval letter for this study and the informed consent form as supporting information.

Secondly, we have completed the all items on the Human Participants Research Checklist that are relevant for our submission.

Thirdly, we have removed the figures from the manuscript file and kept the captions of these figures in the manuscript, to ensure the figures are placed at the right sections of the document.

We hope these actions meet the requirements.

Regards

Sehar un Nisa Hassan, Ph.D.

Corresponding author
