# Supplementary material for: Undergraduates’ lifestyle and Suboptimal Health Status (SHS): A cross-sectional study in the Ha’il region of Saudi Arabia
Source: PLoS One. 2025 Jan 24;20(1):e0317127. doi: 10.1371/journal.pone.0317127 (PMC11761099; doi:10.1371/journal.pone.0317127)
Supplement: Attachment — Submitted filename: Response to reviewers comments.pdf [file pone.0317127.s003.pdf]

Subject: Response to the Editor and Reviewers' Comments on research article submitted for consideration for publication at PLOS ONE.

Original Research Article

PONE-D-24-20915

Undergraduates' lifestyle and suboptimal health status (SHS): A cross-sectional study in the Ha'il region of Saudi Arabia

We thank you for the review and valuable comments on our manuscript. Please find below the point-by-point response to both editorial and reviewers' comments. We hope for your kind consideration.

Editorial Requirement 1: A rebuttal letter that responds to each point raised by the academic editor and reviewer(s). You should upload this letter as a separate file labelled *'Response to Reviewers'*.

Response 1: Done.

Editorial Requirement 2: A marked-up copy of your manuscript highlighting changes made to the original version. You should upload this as a separate file labelled 'Revised Manuscript with Track Changes'.

Response 2: The manuscript with changes highlighted in yellow has been submitted as a separate file.

Editorial Requirement 3: An unmarked version of your revised paper without tracked changes. You should upload this as a separate file labelled 'Manuscript'.

Response 3 : An unmarked copy of the manuscript has been uploaded as well.

Editorial Requirements 4: Please ensure that your manuscript meets PLOS ONE's style requirements, including those for file naming.

Response 4:The manuscript has been transformed according to the template requirements as per guidelines provided in the templates.

Editorial Requirements 5: Please state what role the funders took in the study. If the funders had no role, please state: "The funders had no role in study design, data collection and analysis, decision to publish, or preparation of the manuscript.""

If this statement is not correct you must amend it as needed.

Response 5: Yes, the funder has no role, and the required adjustment has been made in the funding statement.

Editorial Requirement 6: Data requests to a non-author institutional point of contact, such as a data access or ethics committee, help guarantee long term stability and availability of data. Providing interested researchers with a durable point of contact ensures data will be accessible even if an author changes email addresses, institutions, or becomes unavailable to answer requests.

Response 6: Thank you for this important point. We agree that appropriate measures must be taken to guarantee long term stability and availability of data. We have uploaded the anonymous copy of data set on Kaggle as well have provided a non-author institutional point of contact as well in the data availability statement.

Access to anonymous data used for this analysis is submitted along with this article and is available at https://www.kaggle.com/datasets/drseharunnisahassan/lifestyle-and-suboptimal-health/data. Further information about data can be obtained upon direct request sent via e-mail to either the corresponding author on this manuscript or the secretary of training at the College of Public Health and Health Informatics at: training.cphhi@uoh.edu.sa

Reviewers Comments

Response to Reviewer 1 Comments

Comment #1: Please write the nomenclature of SHS in the abstract. The conclusion needs to be rephrased in accordance with the objectives.

Response 1: Adjusted the changes. (Pg 3; Line 35). Rephrased the conclusion (Pg 3 Line 46-48).

Comment # 2: Add future research focus on intervention study: Future research could explore the effectiveness of interventions aimed at promoting healthy lifestyles and reducing SHS among university students. Qualitative research: Qualitative research could provide deeper insights into the reasons behind students' sedentary behaviours and identify potential barriers to adopting healthy lifestyles.

Response 2: Added the directions for future research as per the above recommendation by the reviewer 1 (Pg 15 Line 319-321).

Response to Comments by Reviewer 2

Comment #1: It's a very well-written manuscript. I would like to congratulate the team for their great work on a very essential area. This can be replicated in other regions and countries to add valuable insights and contribute to academia.

Response #1: Thank you for your appreciation. We have added the point recommended by you in limitations and directions for future research. (Pg 15 Line 317-321).

Response to Comments by Reviewer 3

Comment 1 (Part 1): The manuscript does not offer a theoretical contribution to how we think about population health. It also doesn't offer substantive insights (the main contribution is the implementation of SHSQ-25 in Saudi Arabia). The data presented is correlational, meaning it cannot be used to draw causal inferences.

Response (Part 1): Thank you for this important comment and appreciate it. We made some modifications in the introduction and have added a section with some relevant points on the theoretical framework on population health and SHS (Pg 4; Line 67-81)

Response (Part 2): Appreciate your observation and agree that the study is correlational and drawing causal inferences is not asserted in this manuscript. The title also refers to the current research as a cross-sectional study, however, the regression models can be used to do the predictive analysis based on this cross-sectional data.

Comment 2: Data - I understand the restrictive nature of the data used in this investigation, the ongoing crisis the scientific community has been dealing with in recent years necessitates transparency, encompassing all aspects related to methods, data, and analyses. Having reviewed enough manuscripts, I know it is possible to uphold transparency while protecting participants, companies, and data.

Response 2: Thank you for this important comment. We have added in the data availability statement that now make it possible to access anonymous data directly from the dataset repository or through the institution (Pg 16; Line 333-336). Following data availability statement has been added.

Data Availability Statement: Access to anonymous data used for this analysis is submitted along with this article and available at https://www.kaggle.com/datasets/drseharunnisahassan/lifestyle-and-suboptimal-health/data. Further information about data can be obtained upon direct request sent via e-mail to either the corresponding author on this manuscript or the secretary of training at the College of Public Health and Health Informatics at: training.cphhi@uoh.edu.sa

Comment 3: Health, both emotional and physical, is important across income and education levels and ages. As such, I find the authors' motivation for this investigation–focusing on college students–unfortunate. Even if the data is limited to a certain age group, the authors could probably consider a broader target population. If I'm wrong, and there is something special about college students, the authors should offer compelling arguments.

Response 3. Thank you very much for this important comment. We agree that income, education and age are important determinants of health. This project was carried out as part of the bigger project that has collected data from target populations including the general population with a wide range of age, education and income levels. However, in this analysis, we particularly focused on university students for the following reasons which we have mentioned and further elaborated in the manuscript (Pg 3; Line 51-54); (Pg 5-6; Line 108-119).

Comment 4: Finally, describing SHSQ-25 as a "…well-known self-report measure that has been well-tested for its psychometric properties and has been widely used in the assessment of SHS" is somewhat inaccurate. A search in Google Scholar produced only 209 citations for the original paper (2009), some of which were self-citations.

Response 4: Thank you for this important point. We have rephrased this part of the manuscript in light of your comments. (Pg 7 ; Line 147-149).
